# Immune-related potential biomarkers and therapeutic targets in coronary artery disease

**DOI:** 10.3389/fcvm.2022.1055422

**Published:** 2023-01-06

**Authors:** Chaosheng Liu, Jifeng Liu, Yunshu Zhang, Xi Wang, Yue Guan

**Affiliations:** ^1^Department of Cardiology, The First Affiliated Hospital of Dalian Medical University, Dalian, Liaoning, China; ^2^Department of General Surgery, The First Affiliated Hospital of Dalian Medical University, Dalian, Liaoning, China; ^3^Department of Traditional Chinese Medicine, The First Affiliated Hospital of Dalian Medical University, Dalian, Liaoning, China; ^4^Department of Critical Medicine, The First Affiliated Hospital of Dalian Medical University, Dalian, Liaoning, China; ^5^Department of Cardiology, The Third Affiliated Hospital of Dalian Medical University, Dalian, Liaoning, China

**Keywords:** coronary artery disease (CAD), immune-related genes (IRGs), optimal feature genes (OFGs), bioinformatics analysis, Gene Expression Omnibus (GEO)

## Abstract

**Background:**

Coronary artery disease (CAD) is a complex illness with unknown pathophysiology. Peripheral biomarkers are a non-invasive method required to track the onset and progression of CAD and have unbeatable benefits in terms of early identification, prognostic assessment, and categorization of the diagnosis. This study aimed to identify and validate the diagnostic and therapeutic potential of differentially expressed immune-related genes (DE-IRGs) in CAD, which will aid in improving our knowledge on the etiology of CAD and in forming genetic predictions.

**Methods:**

First, we searched coronary heart disease in the Gene Expression Omnibus (GEO) database and identified GSE20680 (CAD = 87, Normal = 52) as the trial set and GSE20681 (CAD = 99, Normal = 99) as the validation set. Functional enrichment analysis using protein-protein interactions (PPIs), Gene Ontology (GO), and Kyoto Encyclopedia of Genes and Genomes (KEGG) was carried out on the identified differentially expressed genes. Optimal feature genes (OFGs) were generated using the support vector machine recursive feature elimination algorithm and the least absolute shrinkage and selection operator (LASSO) algorithm. Furthermore, immune infiltration in CAD patients and healthy controls was compared using CIBERSORT, and the relationship between immune cells and OFGs was examined. In addition, we constructed potential targeted drugs for this model through the Drug-Gene Interaction database (DGIdb) database. Finally, we verify the expression of S100A8-dominated OFGs in the GSE20681 dataset to confirm the universality of our study.

**Results:**

We identified the ten best OFGs for CAD from the DE-IRGs. Functional enrichment analysis showed that these marker genes are crucial for receptor-ligand activity, signaling receptor activator activity, and positive control of the response to stimuli from the outside world. Additionally, CIBERSORT revealed that S100A8 could be connected to alterations in the immune microenvironment in CAD patients. Furthermore, with the help of DGIdb and Cytoscape, a total of 64 medicines that target five marker genes were subsequently discovered. Finally, we verified the expression of the OFGs genes in the GSE20681 dataset between CAD patients and normal patients and found that there was also a significant difference in the expression of S100A8.

**Conclusion:**

We created a 10-gene immune-related prognostic model for CAD and confirmed its validity. The model can identify potential biomarkers for CAD prediction and more accurately gauge the progression of the disease.

## 1. Introduction

In coronary artery disease (CAD), myocardial ischemia, hypoxia, and necrosis are the result of narrowing or obstruction of the lumen caused by atherosclerotic lesions of the coronary arteries (1, 2). CAD is one of the deadliest diseases worldwide, with 12 million people per year expected to die from CAD by 2030 (3). The morbidity and death rates associated with CAD are rising in low- and middle-income countries and are now on par with those in developed nations, making it an issue worldwide (3, 4). Atherosclerosis progresses slowly over decades and due to sub-typical symptoms, the start of CAD is frequently overlooked (5). The pathogenesis of CAD is complicated and remains to be fully understood. Although coronary angiography is the most effective method for diagnosing CAD, it is intrusive and expensive. To develop predictive, diagnostic, or prognostic tools for CAD, more combinations of biomarkers must be included utilizing various techniques. Over time, atherosclerosis is suggested to be a lipid storage disease; however, it is becoming increasingly clear that inflammation links dyslipidemia and other risk factors to atherosclerosis formation, which ultimately leads to the formation of coronary heart disease (6). Therefore, it is highly feasible to analyze differences in the expression of immune-related genes (IRGs) between CAD patients and healthy controls to identify and validate the diagnostic and therapeutic in CAD.

CAD patients frequently experience changes in the immunological components of their peripheral blood throughout their illness, which have been linked to acute exacerbations, remission, and stability of the disease (7, 8). Thus, these immunological components can also be used as therapeutic targets or biomarkers for disease monitoring. The secondary prevention of CAD depends on accurate detection and subsequent mechanistic investigation into these immunological components. The pathophysiology and development of CAD are thought to be significantly influenced by immunological diseases (9). Before the onset of the pathogenic phenomena, specific immunological changes occur (10). However, as the result of the current sample size and project scope remain constrained, these novel targets are rarely verified or mechanistically investigated further, which restricts their translation to the clinic (11, 12). Therefore, despite compelling data, further research is needed to fully understand the peripheral immunological characteristics and processes of CAD.

From the perspective of the primary prevention of CAD, our concern is whether new risk markers for atherosclerosis can improve CAD risk prediction. However, existing biomarkers, including heat shock C-reactive protein (hsCRP), are intertwined with inflammation, oxidation, hemostasis, and other processes involving atherosclerosis, and the results are not ideal (13). In addition, many histopathologies have also shown that inflammation is closely related to CAD, and monocytes and mast cells play a crucial role in the chronic inflammatory response (14). Therefore, it is of clinical significance to establish an immune-related prognostic model to identify and validate the diagnostic and therapeutic in CAD. In this study, we developed a new diagnostic model of optimal feature genes (OFGs) using differentially expressed immune-related genes (DE-IRGs) in CAD patients. We then performed correlation analysis and testing, which demonstrated that the model, particularly the S100A8 gene, has strong potential as a target for diagnostic and therapeutic interventions. This study aimed to identify and validate the diagnostic and therapeutic potential of DE-IRGs in CAD, which will aid in improving our knowledge on the etiology of CAD and in forming genetic predictions.

## 2. Materials and methods

### 2.1. Data preparation

Gene expression information for CAD and standard samples was retrieved from the Gene Expression Omnibus (GEO) database. The GSE20680 dataset, which has 139 models overall and includes 52 standard samples and 87 CAD samples, was used as the training set for the study’s principal analysis (15). We then verified the expression of marker genes using the GSE20681 dataset. The Immunology Database and Analysis Portal (immPort) database provided the 1793 IRGs (Supplementary Table 1) used in this study (16). Drugs that target marker genes were predicted using the Drug-Gene Interaction Database (DGIdb) (17). Additionally, the DrugBank database was used to collect structural data on drugs that target marker genes.

### 2.2. Identification of DE-IRGs

We screened for DE-IRGs between CAD and controls using the “LIMMA” package in the R software using the combined datasets (*P* < 0.05). Genes were categorized as being up-regulated or down-regulated based on their log2FC values (18), which are displayed on a Volcano plot.

### 2.3. Functional enrichment analysis

Many related human pathologies, including cancer, neurodegenerative diseases, and infectious diseases, are the result of abnormal protein-protein interactions (PPIs) that alter molecular recognition mechanisms and binding partner affinity under given conditions. Highly complex interactome diagrams are increasingly being used to decipher disease-specific protein associations and characterize the effects of splicing and genetic variation on these systems (19). We confined the confidence (combined score) > 0.4 as a condition when examining the PPI to establish the validity of this interaction.

The Gene Ontology (GO) project provides a comprehensive source for functional genomics. There are three separate aspects: (i) development and maintenance of the ontology, (ii) annotation of gene products, and (iii) development and continuous improvement of tools and training that facilitate the creation, maintenance, and use of the ontologies (20). The Kyoto Encyclopedia of Genes and Genomes (KEGG) is a knowledge base for the systematic analysis of gene functions, linking genomic information with higher order functional information. The PATHWAY database represents the higher order functions in terms of the network for the interacting molecules (21). As a result, with the help of the “ClusterProfiler” package in R, GO was used to identify characteristic biological attributes, and KEGG pathway enrichment analysis was performed to identify functional attributes. Significance was set at *P* < 0.05.

### 2.4. Identifying CAD-related OFGs

In biomedical research, it is important to select the variables most associated with the studied outcome and to determine the strength of this association. Least absolute shrinkage and selection operator (LASSO) Cox regression analyses and support vector machines-recursive feature elimination (SVM-RFE) analyses are powerful tools to analyze data with a number of predictors approximately equal or larger than the number of observations. The “glmnet” package was used to obtain the LASSO algorithm to minimize the dimensionality of the data. By locating distinct genetic biomarkers of CAD using the LASSO technique, which were compared by the average misjudgment rates of their 10-fold cross-validations, models of DE-IRGs between CAD patients and normal samples were created (22). A SVM-RFE model was created at the same time using the “SVM” package, and its average false positive rate was also compared using a 10-fold cross-validation (23). To create OFGs for CAD, genes from both algorithms were overlapped. By determining the receiver operating characteristic (ROC) curve and computing the area under the curve (AUC), accuracy, sensitivity, and selectivity, the diagnostic potency of OFGs was evaluated. Similarly, the diagnostic power of the logistic regression model was evaluated using ROC curves.

### 2.5. Single sample gene set enrichment analysis (ssGSEA) and single-gene gene set variation analysis (ssGSVA)

Gene set enrichment analysis (GSEA) is a popular framework for condensing information in gene expression profiles into pathways or signature summaries. While GSEA is often considered the endpoint of bioinformatics analysis, gene set variation analysis (GSVA) has an increased ability to detect subtle pathway activity changes in sample populations compared to GSEA, which constitutes the starting point for building pathway-centric biological models (24). The background gene set for this study was the KEGG pathway set, and each marker gene underwent GSVA analysis using the “GSVA” package in the R software. Simultaneously, we utilized the “LIMMA” package to examine the variations in GSVA scores between the marker gene groups with high and low expression. Based on the filtering criteria of |*t*| > 2 and *P* < 0.05, if *t* > 0, we regarded the pathway to be activated in the high expression group, and if *t* < 0, we considered the pathway to be activated in the low expression group. Furthermore, with the help of the “LIMMA” package, we performed Single sample gene set enrichment analysis (ssGSEA) to further understand the functions of the OFGs.

### 2.6. Evaluation of CAD immune cell infiltration

CIBERSORT is a type of deconvolution algorithm, by which the normalized gene expression matrix can be transformed into the composition of infiltrating immune cells. Through the CIBERSORT package, we examined the proportion of 28 infiltrating immune cell types in CAD patients and healthy controls. We presented them as risk heat maps and violin plots to better understand the infiltration of immune cells in the samples in the dataset (25). Additionally, we examined the immune cell infiltration associated with each gene and visualized it as a correlation heatmap (26).

### 2.7. Statistical analysis

R version 4.1.3 was used to complete all analyses. Using Student’s *t*-tests, comparisons between the two groups were made. The association between DE-IRGs was discovered using Pearson correlation analysis. Targeted drugs were displayed using Cytoscape. Differences were deemed significant at *P* < 0.05.

## 3. Results

### 3.1. Recognition of DE-IRGs

**FIGURE 1:**
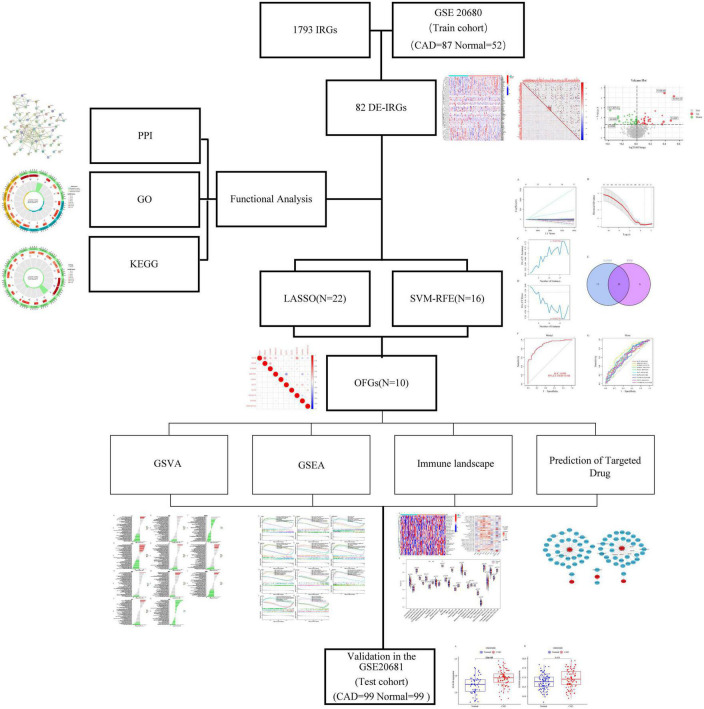
The network of this article.

**FIGURE 2:**
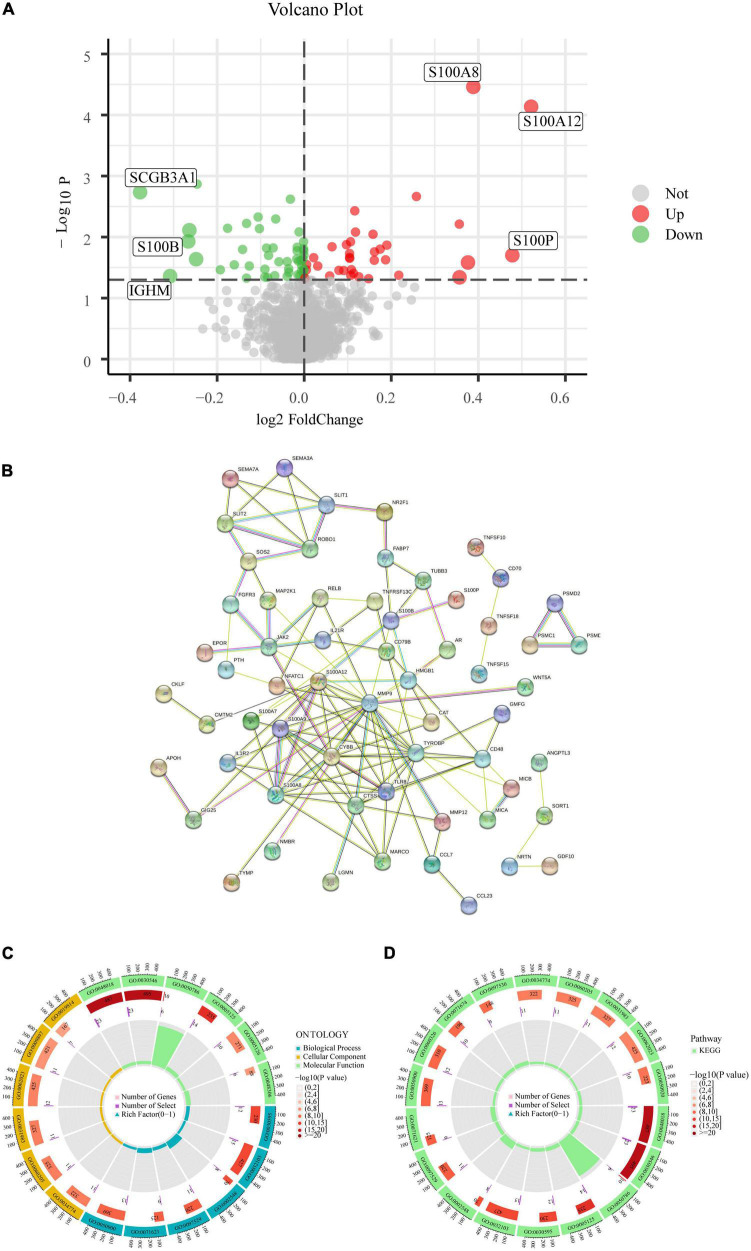
Analysis of DE-IRGs. **(A)** Volcano plot of the DE-IRGs in CAD. **(B)** PPI network. **(C)** GO enrichment analysis. **(D)** KEGG enrichment analysis.

The network is described in Figure 1. With the help of the “LIMMA” package, 82 of the 1,793 IRGs, which consisted of 35 up-regulated and 47 down-regulated genes, were differentially expressed between CAD and healthy controls according to differential analysis of the GSE20680 data (Supplementary Table 2). To show the differential expression of the 82 genes between CAD and control samples, we generated a volcano plot to show genetic differences and marked the three genes that were the most significantly up-regulated and down-regulated (*P* < 0.05) (Figure 2A).

### 3.2. Functional analysis of the DE-IRGs

We built a PPI network to investigate the connection between the IRGs and the biological processes engaged in the regulation process. Of the 82 IRGs, 15 were unrelated, thus, we constructed a PPI network based on the remaining 67 IRGs. These genes were tightly connected at the protein level, according to PPI analysis (Figure 2B). IRGs involved in molecular functions, biological processes, and cellular components were identified by GO analysis; IRGs principally focused on the activation of signaling receptors in the molecular function component, whereas in the biological processes component, they were concentrated on the control of the positive response to stimuli from the environment. Additionally, IRGs were found to be primarily abundant in the outer layer of the plasma membrane in the cellular component aspect (Figure 2C). The GO results were also validated by KEGG; the pathway enrichment map revealed that the primary enriched pathways involved receptor ligand activity, signaling receptor activator activity, and positive response regulation to external stimulus, indicating a strong correlation between the inflammatory response and CAD (Figure 2D).

### 3.3. Identification of OFGs for CAD

**FIGURE 3:**
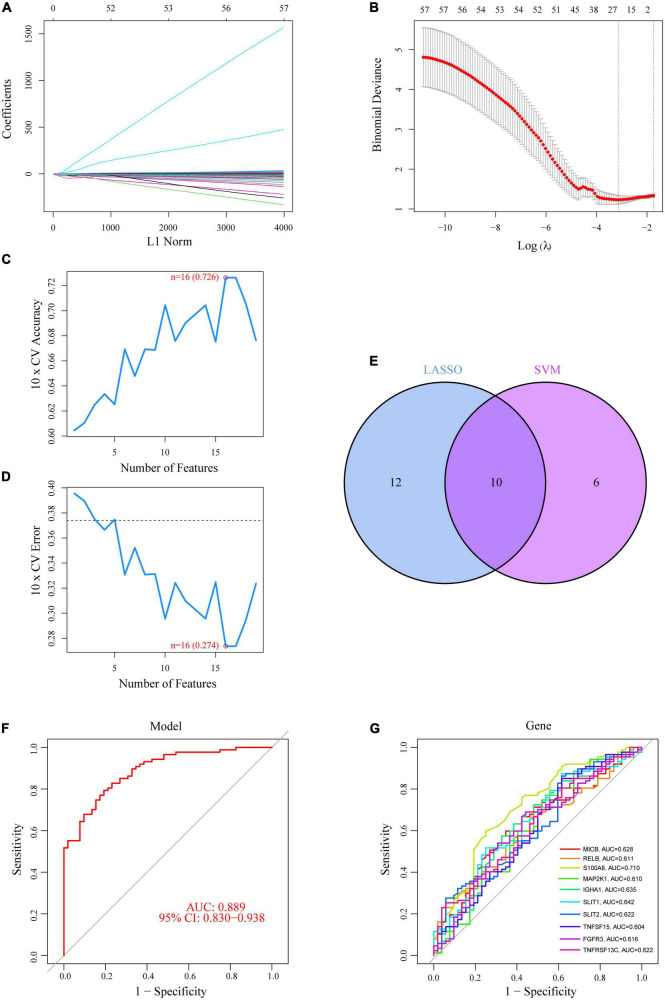
Ten DE-IRGs were shown to be CAD diagnostic genes. **(A,B)** Using the LASSO logistic regression algorithm, with penalty parameter tuning conducted by 10-fold cross-validation, 22 CAD-related features were selected. **(C,D)** The SVM-RFE algorithm was used to filter the 10 DE-IRGs to identify the optimal combination of feature genes. Finally, 16 genes (maximal accuracy = 0.726, minimal RMSE = 0.274) were identified as the OFGs. **(E)** The 10 marker genes obtained from the LASSO and SVM-RFE models. **(F)** The AUC for disease samples was determined using a logistic regression model. **(G)** ROC curves for the 10 marker genes.

To reduce the contingency of the DE-IRGs, we created IRG models sequentially using the LASSO and SVM-RFE approaches, and chose the intersection genes of the two as OFGs to screen out DE-IRGs between CAD patients and normal controls. Based on the average false positive rate of 10 times cross-validation, we created a model consisting of 22 DE-IRGs by applying the LASSO logistic regression algorithm and tweaking the penalty value using 10-fold cross-validation (Figures 3A, B). Then, using the SVM-RFE approach, which was compared by the average misjudgment rates of their 10-fold cross-validations, we created a model including 16 DE-IRGs, which was then used to determine the optimal combination of eigengenes (maximal accuracy = 0.726, minimal RMSE = 0.274) (Figures 3C, D). Finally, we combined the marker genes from the LASSO and SVM-RFE models to identify 10 marker genes (MICB, RELB, S100A8, MAP2K1, IGHA1, SLIT1, SLIT2, TNFSF15, FGFR3, and TNFRSF13C) for further investigation (Figure 3E).

Using the “glm” package, we created a logistic regression model based on the 10 OFGs mentioned above, which could distinguish between normal and CAD samples with an AUC of 0.889 [95% confidence interval (CI): 0.830–0.938], according to the ROC curve (Figure 3F). Furthermore, ROC curves for the 10 marker genes were created to clarify how well a single gene can distinguish between samples with and without CAD; all genes exhibited an AUC > 0.6, with the S100A8 gene having the best predictive power (AUC = 0.710) (Figure 3G). Logistic regression models are more accurate and specific than individual marker genes at differentiating CAD samples from healthy controls.

### 3.4. ssGSVA and ssGSEA

**FIGURE 4:**
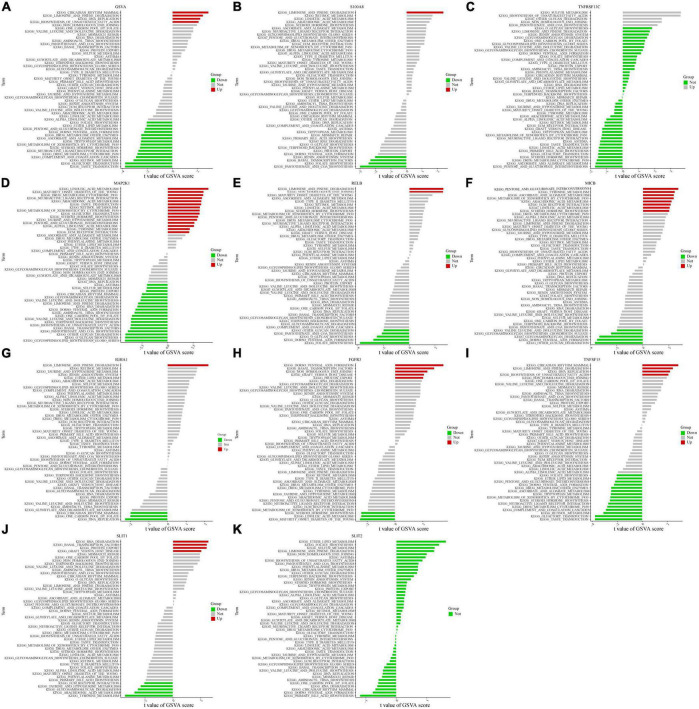
Single-gene GSVA. **(A)** OFGs model of GSVA results. **(B–K)** Expression levels of single marker genes in the GSVA.

Based on the levels of expression of each marker gene paired with GSVA, we found differentially activated pathways across groups with high and low expression. We began by running GSVA analysis on OFGs; CAD patients exhibited lower expression in “taste transduction,” “olfactory transduction,” and “retinol metabolism” compared to normal controls, and increased expression in “circadian rhythm mammalian,” “limonene and pinene degradation,” and “DNA replication” (Figure 4A). Based on the degree of each marker gene’s expression in combination with GSVA, we could identify differences between CAD and normal controls. The expression of the IGH1 gene was mainly up-regulated for “limonene and pinene degradation,” whereas it was down-regulated for “DNA replication.” Patients with CAD exhibited upregulation of the MAK2P1 gene in “linoleic acid metabolized” and downregulation in “glycosphingolipid biosynthetic Globo series.” Patients with CAD had higher levels of “pentose and glucuronic acid interconversion” and lower levels of “other glycan degradation” for the MICB gene. For the RELB gene, “limonene and pinene degradation” and “folate biosynthesis” were the two key areas where it was up-regulated. In addition to being up-regulated in “limonene and pinene degradation,” the S100A8 gene was also down-regulated in “pantothenic acid and COA biosynthesis.” RNA degradation and tyrosine metabolism for the SILT1 gene were both increased in CAD patients. TNFSF15 was downregulated in “taste conduction” but upregulated in “mammalian circadian rhythm.” Additionally, TNFRSF13C was exclusively up-regulated in “sulfur metabolism,” “biosynthesis of unsaturated fatty acids,” “non-homologous end joining,” and “pantothenic acid and COA biosynthesis.” The values were different for other aspects but were not statistically significant. Surprisingly, there was no statistically significant distinction for the SILT2 gene between CAD patients and healthy individuals (Figures 4B–K). From the above GSVA analysis, it is not difficult to conclude that the higher expression of these genes in coronary heart disease patients is related to cell cycle activity and energy metabolism, which to a certain extent reflects the correlation between coronary heart disease and the chronic inflammatory response.

To further explore the potential function of marker genes that can distinguish diseased samples from normal samples, we performed GSEA analysis on the OFGs model and drew the enrichment curve for the top six signaling pathways. The results showed that for CAD patients, the “B cell receptor signaling pathway,” “chronic myeloid leukemia,” “endometrial cancer,” and “lysosome” pathways were up-regulated, while the “neuroactive ligand-receptor interaction” and “olfactory conduction” pathways were down-regulated (Supplementary Figure 1).

### 3.5. Immune landscape

**FIGURE 5:**
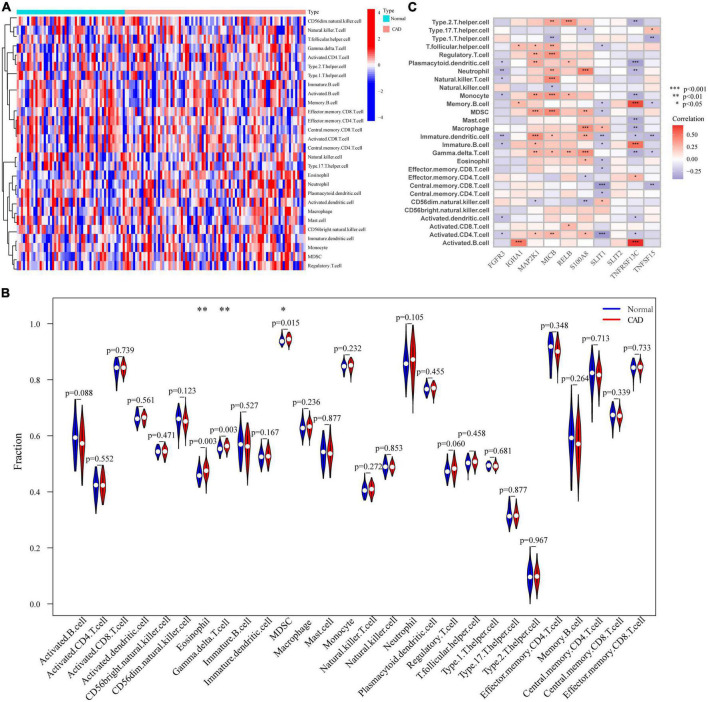
Analysis of the immunological landscape. **(A)** Immune cell expression levels in samples from healthy individuals and CAD patients. **(B)** Comparison of immunological microenvironments in CAD patients and healthy samples. **(C)** Differential study of the immunological microenvironments caused by single genes in OFGs. *p < 0.05, **p < 0.01, ***p < 0.001.

There is increasing data to demonstrate how closely related CAD and the immune microenvironment are. To investigate changes in the immune microenvironment between CAD patients and normal controls, we utilized the CIBERSORT method; after examining the expression levels of 28 types of immune cells in various patient populations 13 were comparatively up-regulated and 15 were down-regulated in CAD patients. The findings were shown as a heat map (Figure 5A). In addition, we categorized the genes by single-gene immune cell infiltration; IGHA1, MAP2K1, MICB, RELB, and S100A8 were primarily linked to up-regulation of immune cell expression, whereas FGFR3, SILT1, SILT2, TNFRSF13C, and TNFSF15 down-regulated immune cell expression (Figure 5B). We analyzed the difference in the expression level of immune cells in 28 patients with CAD and normal controls, and the results are shown as violin diagrams. Eosinophils, gamma delta T cells, and myeloid-derived suppressor cells were up-regulated in CAD patients, and their distinctions were then shown using correlation analysis with the 28 immune cells. Although other immune cells were also found to differ statistically, it was not significant (Figure 5C).

### 3.6. Prediction of targeted drugs

**FIGURE 6:**
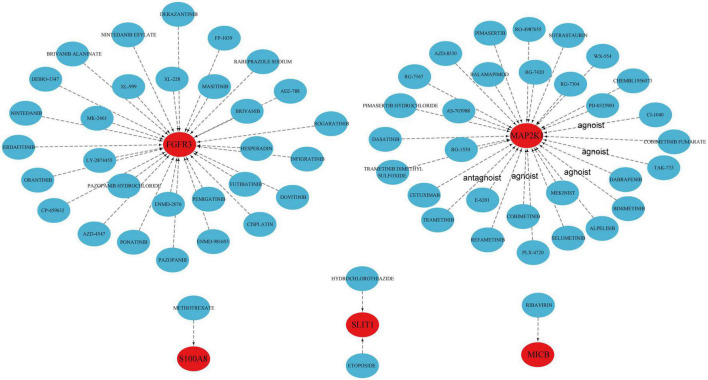
Prediction of marker gene-targeted drugs.

We further revealed the drugs that may target marker genes through the DGIdb database. In total, 64 drugs that were linked to five genes (S100A8, SLIT1, MICB, FGFR3, MAP2K1) were analyzed, including 42 targeted inhibitor drugs, six inhibitor and allosteric modulator drugs, one inhibitor and antagonist drug with controversial effects, and 15 as yet undetermined drugs (Supplementary Table 3). Unfortunately, for the other five genes, we did not find relevant drugs. The results were visualized by the Cytoscape software (Figure 6). Methotrexate (MTX) appears to affect S100A8; however, the precise impact still needs to be validated.

### 3.7. Expression of marker genes in the validation set

**FIGURE 7:**
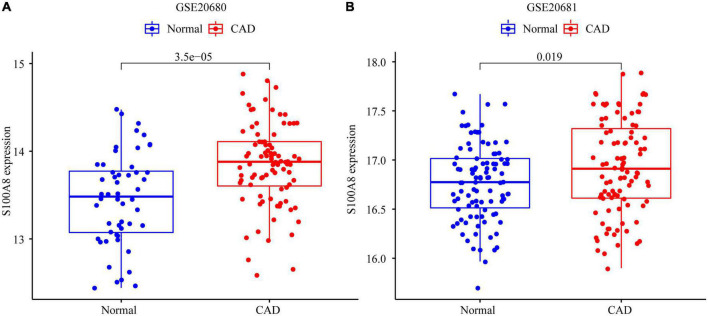
Expression of S100A8 gene in the test set and validation set. **(A)** S100A8 expression in GSE20680. **(B)** S100A8 expression in GSE20681.

In the GSE20681 dataset, we verified the expression of the marker genes. We used differential analysis to examine S100A8 gene expression in the validation set; the results revealed that S100A8 expression was higher in CAD patients than in healthy controls (*P* = 0.019) (Figure 7).

## 4. Discussion

Coronary atherosclerosis is a complex, persistent, and progressive inflammatory disease that is the leading cause of death in both developed and developing countries (2). Immune cells maintain homeostasis in the heart, and various immune cells that reside or penetrate the heart tissue play an important role in the repair process following tissue damage. Many previous studies have suggested that CAD and immunoreaction are inseparable and that inflammation is closely related to CAD; in particular, monocytes and mast cells play a crucial role in the process of the chronic inflammatory response. However, it is worth noting that the specific relationship between coronary atherosclerosis and immune genes has not been reported in detail. Therefore, it is of clinical significance to establish an immune-related prognostic model. Therefore, we used the GSE20680 gene set as original data to analyze the DEGs between CAD patients and normal controls. Next, we found common functional pathways through bioinformatics enrichment, and analyzed the differences in the expression of IRGs between CAD patients and controls, which is of great significance in exploring the pathogenesis of IRGs in CAD patients.

An individual’s risk of CAD is controlled by interactions between hereditary and lifestyle factors, as is the case with most complicated diseases (27). There are several well-acknowledged risk factors for CAD, such as tobacco use, hypertension, hyperlipidemia, diabetes, and family history; at least one of these risk variables is present in 80–90% of patients with documented CAD (28). The study of gene expression, mRNA, and miRNA has opened up new possibilities for the pursuit of diagnostic and therapeutic approaches, widening the boundaries of genetic research as laboratory technology and informatics advance (7). Metabolic syndrome is defined as central obesity plus hypertriglyceridemia, low high-density lipoprotein cholesterol, hypertension, or high fasting glucose levels. Grayson et al. have identified gene expression patterns associated with metabolic syndrome, which includes many genes involved in the innate immune response. As the condition develops into sequelae, such as CAD, which is characterized by an increase in genes related to macrophage activation and signaling, this pattern changes (29, 30). Therefore, immune expression research can be used to infer the stage and prognosis of CAD patients.

Numerous immunological components, such as cells (endothelial cells, macrophages, and lymphocytes), cytokines, chemokines, acute-phase proteins, and adhesion molecules, are involved in atherosclerosis (31). Prospective epidemiological data is most consistent with the predictive usefulness of CRP, interleukin 6 (IL-6), and tumor necrosis factor-alpha (TNF-α), among a wide range of immune system parameters (32). In addition, immune system characteristics are closely related to CAD risk factors, including hypertension, smoking, dyslipidemia, etc. CAD risk factors indirectly induce inflammatory responses to promote the occurrence and development of coronary heart disease. The vicious cycle of inflammation and high-risk factors intertwines and promotes the occurrence of CAD. The initial inflammatory response to arterial injury is frequently advantageous; however, if the harmful agent continues to exist, it could cause permanent inflammation and change the way the body reacts initially to harmful inflammation. The inflammatory process becomes the first feature of the development of CAD, with subsequent progression of inflammation that eventually impairs arterial function (33, 34). Therefore, it is particularly important to identify the early inflammatory response in CAD.

In this study, we screened 10 IRGs, dominated by S100A8, by bioinformatics. Each of these 10 genes has an AUC value > 0.6, indicating that they are accurate and specific at differentiating coronary heart disease samples from normal samples. The S100 family member S100A8 (also known as MRP8) typically appears as a heterodimer. As a Ca^2+^ sensor also involved in cytoskeletal reorganization and arachidonic acid metabolism, S100A8 is constitutively expressed in neutrophils and monocytes. In disorders connected to inflammation, S100A8 has been proposed as a biomarker for diagnosis and follow-up, and as a predictor of therapeutic response (35). S100A8 is actively produced during inflammation and is essential for the inflammatory response since it promotes leukocyte recruitment and triggers cytokine secretion (36, 37). S100A8 controls the production of pro-inflammatory mediators, including cytokines, chemokines, reactive oxygen species, and nitric oxide, among others, to prevent tissue damage brought on by excessive inflammation (38). Therefore, S100A8 expression is a double-edged sword; on one hand, as an inflammatory mediator, it promotes the progression of local inflammation, whereas on the other hand, overexpression of S100A8 will inhibit the explosive spread of inflammation, and eventually lead to the chronic progressive development of inflammation. Clinical evidence suggests that the neutrophil-to-lymphocyte ratio is one of the most reliable predictors of death in acute coronary syndrome (ACS) patients, meaning that lowering the neutrophil count may have positive effects (39, 40). In ACS patients, neutrophils pass through the injury site and release a significant amount of S100A8, which acts as a chemoattractant to encourage the recruitment of other cell types, including monocytes, which determines the subsequent inflammatory response. Additionally, neutrophils are the first responders to aseptic injury (41, 42). Our study found that CAD patients have a high expression of S100A8, which supports the view that a chronic inflammatory response occurs in CAD; therefore, this model may provide a new therapeutic target for the diagnosis and treatment of CAD.

To our knowledge, this is the first study to use bioinformatics to comprehensively analyze the role of IRGs in CAD. In addition, most of the existing studies have constructed prognostic models through methods such as Weighted Gene Co-Expression Network Analysis (WGCNA) (42–45). Compared with them, we are the first to use LASSO and SVM-RFE dual methods to establish a prognosis model of coronary heart disease, which greatly improved the accuracy of the model, which is undoubtedly of certain value. Besides, on the basis of PPI network, GO and KEGG enrichment analysis of differential genes, we further analyzed the single genes of the model prognosis genes, and further demonstrated the specific mechanism of action and related pathways of the model-related genes by using GSVA and ssGSEA methods, which is undoubtedly of constructive significance. Nonetheless, this investigation is not without its drawbacks. First, even though the OFGs had a significant prognostic impact in both the experimental and control groups, we cannot rule out the possibility that the prognostic effect was accidental, thus, it needs to be confirmed in a study with a larger sample size. However, there are currently no data sets with a large enough sample size and clinical prognostic information to be used for further verification. The interaction of other characteristics with predictive value was not considered in this study, even though this OFG model performed exceptionally well in terms of prognosis for patients with CAD. Third, since this prognostic signature was developed and tested using information from open sources, the model requires additional biological support, which will need to be confirmed in subsequent research.

## 5. Conclusion

We formed an immune-associated coronary heart disease prognostic model for a total of 10 genes (MICB, RELB, S100A8, MAP2K1, IGHA1, SLIT1, SLIT2, TNFSF15, FGFR3, and TNFRSF13C). Among them, S100A8 was the most closely related to CAD. Through bioinformatics correlation analysis, we confirmed that the cardiac immune microenvironment of CAD patients may be affected by this OFG model. In addition, we found that the relationship between immune cells and these key genes could have a significant impact on the development and progression of CAD, and that studies of these genes may shed new light on how to treat cardiovascular disease. Although gene expression levels may not always correlate to protein expression, their research importance is indisputable. To better understand the pathophysiology and management of coronary heart disease, we will continue to focus on these genes in future studies.

## Data availability statement

The datasets presented in this study can be found in online repositories. The names of the repository/repositories and accession number(s) can be found below: https://www.ncbi.nlm.nih.gov/geo/, GSE20680 and https://www.ncbi.nlm.nih.gov/geo/, GSE20681.

## Author contributions

All authors were solely responsible for the content and writing of the manuscript and made significant contributions to the design, data collection and interpretation, and manuscript preparation and revision of this study.

## Supplementary material

Supplementary Figure 1 Gene set enrichment analysis (GSEA). **(A)** OFGs model of GSEA results. **(B–K)** Expression levels of single marker genes in the GSEA.

Supplementary Table 1 1793 immune-related genes.

Supplementary Table 2 82 DEGs in GSE20680.

Supplementary Table 3 Marker gene-targeted drugs.
