# Supplementary figures and images for: Immune-related potential biomarkers and therapeutic targets in coronary artery disease
Source: Front Cardiovasc Med. 2023 Jan 6;9:1055422. doi: 10.3389/fcvm.2022.1055422 (PMC9853173; doi:10.3389/fcvm.2022.1055422)

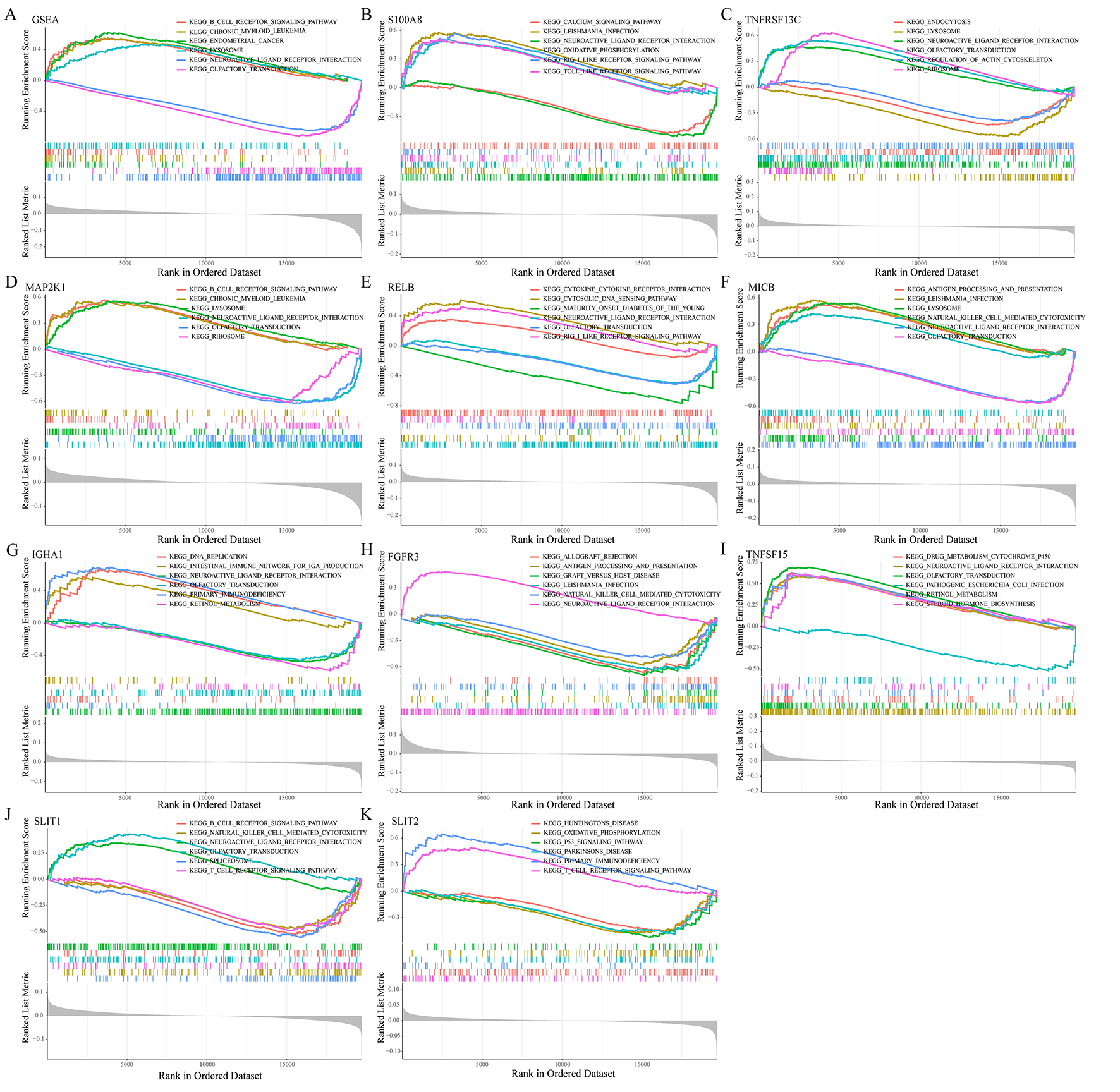

Supplement: Supplementary Figure 1 — Gene set enrichment analysis (GSEA). (A) OFGs model of GSEA results. (B–K) Expression levels of single marker genes in the GSEA. [file Image_1.tif]
